# Sleep physiological network analysis in children

**DOI:** 10.5935/1984-0063.20220022

**Published:** 2022

**Authors:** Alvaro David Orjuela-Cañón, Andrés Leonardo Jutinico, Maria Angelica Bazurto-Zapata, Elida Duenas-Meza

**Affiliations:** 1 Universidad del Rosario, School of Medicine and Health Sciences - Bogota D.C. - Bogota D.C. - Colombia.; 2 Universidad Antonio Nariño, Facultad de Ingeniería Mecánica, Electrónica y Biomédica - Bogota D.C. - Bogota D.C. -Colombia.; 3 Fundación Neumológica Colombiana, Laboratorio del Sueño - Bogota D.C. -Bogota D.C. - Colombia.

**Keywords:** Granger causality, Polysomnography, Brain-heart connectivity, Physiological networks, Heart Rate Variability, Electroencephalography

## Abstract

**Objective:**

Physiological networks have recently been employed as an alternative to analyze the interaction of the human body. Within this option, different systems are analyzed as nodes inside a communication network as well how information fows. Several studies have been proposed to study sleep subjects with the help of the Granger causality computation over electroencephalographic and heart rate variability signals. However, following this methodology, novel approximations for children subjects are presented here, where comparison between adult and children sleep is followed through the obtained connectivities.

**Methods:**

Data from ten adults and children were retrospectively extracted from polysomnography records. Database was extracted from people suspected of having sleep disorders who participated in a previous study. Connectivity was computed based on Granger causality, according to preprocessing of similar studies in this feld. A comparison for adults and children groups with a chi-square test was followed, employing the results of the Granger causality measures.

**Results:**

Results show that differences were mainly established for nodes inside the brain network connectivity. Additionally, for interactions between brain and heart networks, it was brought to light that children physiology sends more information from heart to brain nodes compared to the adults group.

**Discussion:**

This study represents a frst sight to children sleep analysis, employing the Granger causality computation. It contributes to understand sleep in children employing measurements from physiological signals. Preliminary fndings suggest more interactions inside the brain network for children group compared to adults group.

## INTRODUCTION

Sleep is a physiological and reversible state of consciousness that is essential for health. The worldwide prevalence of sleep disorders in the general population is 35%^1^. Sleep quality is related to behavior, attention, memory, emotional regulation, physical health and cognitive functioning in children and adults. Different strategies have been established to assess sleep, such as polysomnography (PSG), videosomnography, actigraphy and subjective reports, which use information from questionnaires, diaries and other data. Of these approaches, researchers seem to prefer methods based on PSG because of its utility for monitoring sleep stages using electroencephalography (EEG) spectral densities^2,3,4^.

Literature in sleep medicine widely agrees that sleep restrictions due to different disorders have adverse effects, including deficiencies in memory, emotional and cognitive processes in children^5,6,7^ and adults^8,9,10^. However, a detailed analysis of the impact of sleep quality and duration in children has been not reported in detail^11^. Therefore, there is a need to employ more tools and analyses that allow for the quantification of sleep activity and its interaction with other processes inside the human body, which will require integrating concepts across disciplines, and to promote the best and new practices in sleep research methods^12^.

In the study of the human physiology, it is possible to represent each functional part of the body as a node, and relationships between those nodes as networks. Based on this idea, an approach has been developed in the past decade in which the communication between different systems, such as the brain, heart, and muscles, is investigated using a physiological networks (PN) concept^13,14^. Using this approach, for example, it is possible to analyze the interaction between the sympathetic and parasympathetic nervous systems through the study of sino-atrial node and heart rate variability (HRV)^15^. This is possible because changes in heart rate (HR) provide information about the autonomic performance of the human system. HRV measures are obtained through the electrocardiographic (ECG) signals, and its following calculation of the RR peaks interval times.

The PN approach makes it possible to study the human body as nodes in a complex system that reflects the dynamic regulation in our bodies^13,14,16^. The study of these networks in relation to the sleep has been reported by Faes et al.^17,18,19^, where the authors investigated information flow between the brain and heart networks in adults during sleep using EEG and ECG recordings from PSG studies. In the studies, the authors examined HRV through a high frequency (HF) band and divided the EEG signal into the δ, θ, α, β and γ bands, and reported a high interaction between the HF power band from HRV and EEG β band. This analysis was possible through the application of the Granger causality (GC) test, which provides a quantification of the communication between nodes^20^.

The previous studies were performed in adult populations. However, it has been reported that sleep differs between children and adults because of differences in brain physiology. Thus, the present study aimed to evaluate the physiological networks in children during sleep, and to compare the results to findings in adults.

## MATERIAL AND METHODS

Data were retrospectively extracted from PSG records from a database of people suspected of having sleep disorders who participated in a PSG study. Two groups were established: i) adults with bruxism, habitual snoring and sleepiness (ages between 25 and 68 years) and ii) children with habitual snoring and breathing pauses during the sleep (ages between 3 and 11 years). All participants were considered as healthy based on Apnea Hypopnea Index (AHI) scores of <10/h for the adults group and ≤2/h for the children group, according to the rules of the American Academy of Sleep Medicine (AASM)^21^. Data from the PSG study were collected in the Sleep Center (CS, by its acronym in Spanish) of the Fundación Neumológica Colombiana (FNC), in compliance with the principles of Good Clinical Practice (GCP). Records were acquired through the Alice 6 LDE system from Phillips ©, during the 2017 – 2018 in Bogota, Colombia.

A technician scored PSG signals, which were labeled by sleep stages in 30-seconds epochs, according to the AASM policies, and were reviewed by a physician expert in sleep medicine. The present study analyzed one EEG channel (C3-M2) and a unique ECG channel (DII) that represented the brain and heart physiological networks, respectively. The EEG and ECG channels were acquired with sample rates of 100 Hz and 200 Hz, respectively, and a resolution with 16 bits.

### Preprocessing

PSG signals were analyzed using GC according to similar studies in this field^19,22,23,24^. The use of the EEG and ECG signals to represent the interaction of the brain and heart networks was carried out through preprocessing to relate the information from these sources. For this, previous studies using PN analysis, specifically Faes et al.^17^ and Jurysta et al.^25^, were used as basis for this step.

To complete the preprocessing, each signal type followed a different path from the time series point of view. EEG signals were linearly detrended and filtered through a Butterworth band-pass with zero-phase and a bandwidth in the interval of 0.01 to 45 Hz. Frequencies within this range were divided into the subbands: δ, 0.5–3 Hz; θ, 3–8 Hz; α, 8–12 Hz; β, 12–16 Hz; and γ, 16–25 Hz. With the support of trapezoidal integration and the fast Fourier transform, the power computation was carried out for each of the subbands. A new time series by subband was estimated through the mentioned calculation using samples from non-overlapping 30-s epochs. In addition, the data were normalized based on the total power for the EEG subbands for whole night. As a result, the brain node and its internal subsystems were represented by five power time series from the EEG system.

Likewise, the HRV for the heart node was determined from processing of ECG signals. First, an upsampling step elevated the sample rate to 400 Hz. Then, a step for R peaks detection was developed based on the Pan–Tompkins algorithm^26^. The obtained RR intervals were upsampled to 8 Hz and subdivided into 300-s windows with an overlap of the last 270 s to compute the power. For this, three subbands were analyzed: very low frequency (VLF), 0.003–0.04 Hz; low frequency (LF), 0.04–0.15 Hz; and high frequency (HF), 0.15–0.4 Hz^27^. The time series extracted from the RR intervals were detrended, Hanning windowed, and passed by the fast Fourier transform to determine the power spectrum. Finally, the data were normalized based on the total power considered for the HRV (0.003–0.4 Hz) for the entire night. As a result, the heart node was represented by three power time series from the HRV subbands

### Interaction computation

We used GC to evaluate the PN interaction through the five time series (brain node) and the three time series (heart node). GC is a measure that quantifies the directional connectivity based on a prediction developed in two or more systems. To exemplify this, we represent the first system or subsystem across the time series with X_1_ and a second subsystem with X_2_. Then, considering X_2_ as the source and X_1_ as the target, X_2_ Granger-causes X_1_ when the use of information from past values of X_2_ improves the prediction of X_1_ in comparison to when this information is not used. For this, a bivariate autoregressive model of order p AR[p] can be described by expressions (1) and (2):


(1) $$X_{1}\left(t\right)=\sum \underset{j=1}{\rho }A_{11,j}X_{1}\left(t-j\right)+\sum \underset{j=1}{\rho }A_{12,j}X_{2}\left(t-j\right)+\varepsilon_{1}\left(t\right)$$

(2) $$X_{2}\left(t\right)=\sum \underset{j=1}{\rho }A_{21,j}\underset{1}{X}\left(t-j\right)+\sum \underset{j=1}{\rho }A_{12,j}X_{2}\left(t-j\right)+\varepsilon_{2}\left(t\right)$$

where X_1_(t) and X_2_(t) are two time series that represent two process components (in our case, an EEG and HRV subband), *ρ* (order of the model) is the maximum number of past observations included in the model, Aj is a matrix with model coefficients for each j=1... *ρ*, and ε_i_ are the residuals of the prediction for each variable.

To quantify the difference of using the source process past, the GC measure proposes two models: a full model that includes information from all subsystems involved, and a reduced model that uses only part of the information. As a prediction is obtained, the error can be computed from each model as ε_*full*_ and ε_*reduced*_. In this way, the GC from *X*_2_ to the process *X*_1_ can be computed by the expression^6^:


(3) $$F_{2\to 1/n}=ln\frac{\left|\varepsilon_{reduced}\right|}{\left|\varepsilon_{full}\right|}$$

where ε_*full*_ and ε_*reduced*_ are the errors for the reduced and full model, respectively.

For the case of more systems, it is straightforward to extend the computation for multivariate autoregressive (MVAR) models, employing covariance matrices instead of the exhibited errors. In the present study, we analyzed eight power time series in total to represent the brain node across its subsystems (α, β, δ, γ, and θ subbands) and the heart subsystems (VLF, LF, and HF subbands). Both groups (children and adults) were compared in terms of the connectivity computations. Power time series were estimated for sleep stages based on non- and rapid eye movement (REM) indicated by the technician expert in sleep medicine.

To obtain the order of the MVAR models, we tested different values from one to twenty, and chose the model with the best error based on the Bayesian information criterion. At the same time, we also took stability of the model into account. The computation of the MVAR coefficients was carried out with the Levinson-Whittle recursion (LWR) algorithm, which is considered as an extension of the Durbin’s recursion^28^. As a result, 56 connectivity computations were obtained with the use of GC measures. Self-connections were excluded because of insertion of noise. The computation was accomplished with the aid of the MVGC toolbox described previously^29^.

### Groups comparison

Two groups were analyzed (children and adults) and compared. For this, connectivity interaction for each subject was represented as a matrix with binary values, where one (1) represented a found connectivity and zero (0) represented absence of this connectivity, given by the GC computation. Subsequently, matrices for each group were summed and normalized by the maximum value (n = 10 subjwects in each group), demonstrating the frequency of each connection between subsystems.

In addition, sleep stages were studied according to light sleep, which consisted of NREM stages 1 and 2, and deep sleep, which consisted of NREM stage 3 and REM sleep. Finally, the groups were compared using the chi-square test with a p-value < 0.05 considered as significant. We performed comparisons for the entire night, for the light sleep stage, deep sleep stage and REM stage.

## RESULTS

Ten adults (25 to 68 years old, mean of 36.2 + 12 y; Table 1) and 10 children (2 to 11 years old, mean 7 + 3 y; Table 2) were assessed for age, sex, AHI, and percentage in each sleep stage. Duration in sleep stages showed a reduction of REM stage sleep from 19% in the children group to 16% in the adults group (see Tables 1 and 2).

**Table 1:** Characteristics of the Adults Group.

Subject	Age	Sex	AHI	Duration (period 30 s)	Light Sleep	Deep Sleep	REM Sleep	Awake
**1**	44	F	3.2	820	478 (58%)	177 (22%)	100 (12%)	65 (8%)
**2**	30	F	0.4	969	446 (46%)	171 (18%)	218 (22%)	134 (14%)
**3**	25	F	3.2	969	467 (48%)	167 (17%)	141 (15%)	194 (20%)
**4**	41	M	4.4	980	529 (54%)	140 (14%)	243 (25%)	68 (7%)
**5**	36	F	3.6	857	316 (37%)	284 (33%)	146 (17%)	111 (13%)
**6**	25	F	1.3	856	331 (39%)	292 (34%)	149 (17%)	84 (10%)
**7**	68	F	6.7	1074	336 (31%)	439 (41%)	194 (18%)	105 (10%)
**8**	35	F	1.0	938	511 (54%)	278 (30%)	65 (7%)	84 (9%)
**9**	28	F	4.2	849	541 (63%)	81 (10%)	134 (16%)	93 (11%)
**10**	30	F	4.4	854	437 (51%)	172 (20%)	77 (9%)	168 (20%)
**Mean**	**36.2**		**3.24**	**916.6**	**439.2 (48%)**	**220.1 (24%)**	**146.7 (16%)**	**110.6 (12%)**

AHI: Apnea Hypopnea Index, REM: Rapid Eye Movement.

**Table 2:** Characteristics of the Children Group.

Subject	Age	Sex	AHI	Duration (period 30 s)	Light Sleep	Deep Sleep	REM Sleep	Awake
1	2	F	0.5	817	301 (37%)	299 (37%)	120 (15%)	97 (11%)
2	4	F	0.4	883	395 (45%)	279 (32%)	154 (17%)	55 (6%)
3	10	M	0.5	966	426 (44%)	252 (26%)	202 (21%)	86 (9%)
4	10	F	0.8	964	436 (45%)	155 (16%)	139 (15%)	234 (24%)
5	9	M	0.3	926	479 (52%)	151 (16%)	196 (21%)	100 (11%)
6	4	F	0.3	903	369 (41%)	205 (23%)	249 (27%)	80 (9%)
7	10	M	0.1	845	512 (61%)	137 (16%)	142 (16%)	54 (7%)
8	5	F	0.0	913	368 (40%)	207 (23%)	252 (28%)	86 (9%)
9	5	F	1.6	1007	471 (47%)	249 (25%)	162 (16%)	125 (12%)
10	11	F	1.8	852	588 (69%)	93 (11%)	145 (17%)	26 (3%)
Mean	7		0.76	907.6	434.5 (48%)	202.7 (22%)	176.1 (19%)	94.3 (11%)

AHI: Apnea Hypopnea Index, REM: Rapid Eye Movement.

**Figure 1:**
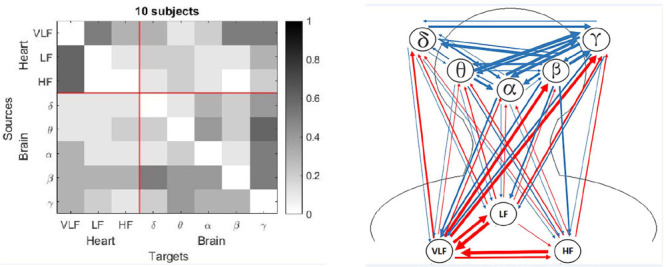
Connections for the Adults group: a) Matrix with normalized values for each connection, and b) Blue rows indicate connections from brain to heart and red rows indicate connections from heart to brain. Thickness of the lines indicates the number of subjects with that connection.

**Figure 2:**
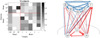
Connections for the Children group: a) Matrix with normalized values for each connection, and b) Blue rows indicate connections from brain to heart and red rows indicate connections from heart to brain. Thickness of the lines indicates the number of subjects with that connection.

**Figure 3:**
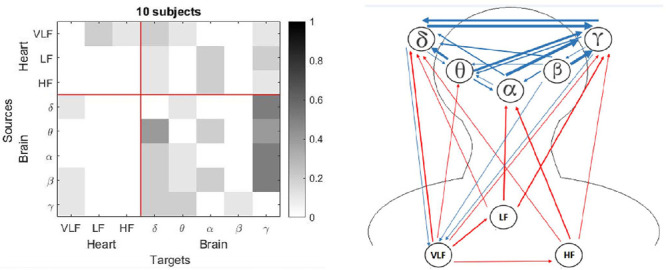
Connections for the adults group for light sleep (non-rapid eye movement [NREM] stages 1 and 2): a) Matrix with normalized values for each connection, and b) Blue rows indicate connections from brain to heart and red rows indicate connections from heart to brain. Thickness of the lines indicates the number of subjects with that connection.

**Figure 4:**
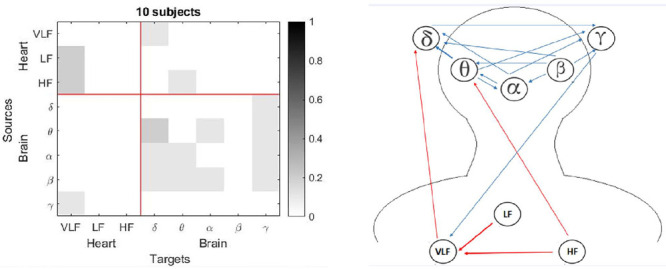
Connections for the adults group for deep sleep (non-rapid eye movement [NREM] stage 3): a) Matrix with normalized values for each connection, and b) Blue rows indicate connections from brain to heart and red rows indicate connections from heart to brain. Thickness of the lines indicates the number of subjects with that connection.

**Figure 5:**
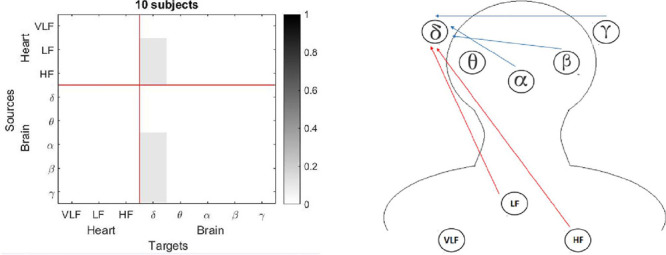
Connections for the adults group for rapid eye movement [REM] sleep: a) Matrix with normalized values for each connection, and b) Blue rows indicate connections from brain to heart and red rows indicate connections from heart to brain. Thickness of the lines indicates the number of subjects with that connection.

**Figure 6:**
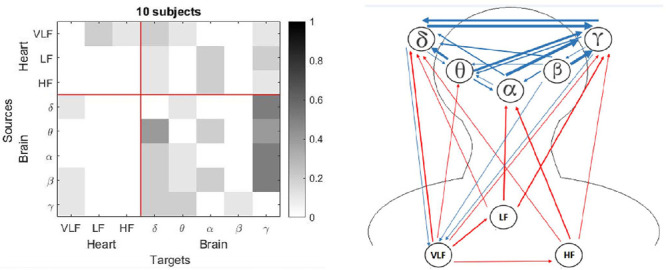
Connections for the children group for the light sleep (non-rapid eye movement [NREM] stages 1 and 2): a) Matrix with normalized values for each connection, and b) Blue rows indicate connections from brain to heart and red rows indicate connections from heart to brain. Thickness of the lines indicates the number of subjects with that connection.

**Figure 7:**
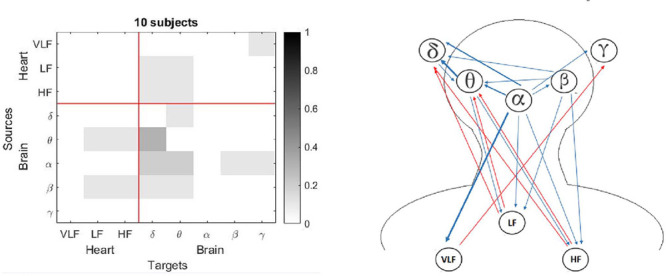
Connections for the children group for the deep sleep (non-rapid eye movement [NREM] stage 3): a) Matrix with normalized values for each connection, and b) Blue rows indicate connections from brain to heart and red rows indicate connections from heart to brain. Thickness of the lines indicates the number of subjects with that connection.

**Figure 8:**
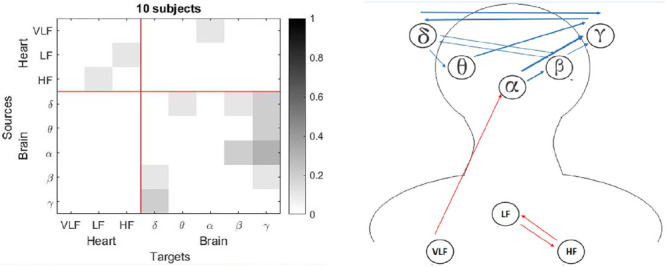
Connections for the children group for the rapid eye movement [REM] sleep: a) Matrix with normalized values for each connection, and b) Blue rows indicate connections from brain to heart and red rows indicate connections from heart to brain. Thickness of the lines indicates the number of subjects with that connection.

The most important finding in the present study was the differences in the brain-heart interactions between children and adults during sleep. The children had increased connectivity from the heart networks towards the brain network (Figs. 1 and 2), mainly for the γ subband, compared with the adults. For the figures 1 to 8, the number of interactions is provided in two different ways: Part a) in each figure shows a matrix subdivided into the brain and heart nodes and their subsystems. Gray tones represent the connectivity obtained for all subjects, where darker tones indicate a higher number of subjects who exhibited that connection. The gradient bar on the right side of the matrix indicates the normalized number of connections for a maximum value of 10 subjects. Part b) visualizes how the connections were established between each subsystem. Blue arrows represent connections from a brain source and red arrows from a heart source. Thickness of the lines indicates the number of subjects with that connection.

The children group (see Fig. 2) had a higher number of connections related to the heart network than the adults group (see Fig. 1) for the sleep entire night, with more red arrows as outputs to the brain network, mostly for the γ subband. Additionally, there were more blue arrows from the brain node to the VLF subsystem.

For the adults group, light sleep, deep sleep and REM sleep presented a reduction of the connectivities between the studied networks (Figs. 4 to 5). Few number of connectivities from heart to brain in adults was seen, visualizing from VLF and HF ones, and with a modest performance inside the heart network for deep sleep (see Fig. 4). REM sleep reported connectivities entering to δ subband from α, β and γ inside the brain network, and from LF and HF from the brain network (Fig. 5). Moreover, for the children group, connectivities for three stages: light sleep, deep sleep and REM sleep presented a notable reduction for the connection from heart to brain (Figs. 6 to 8). For the light sleep this decrement was observed mainly from the LF and HF subbands (see Fig. 6), and for the deep sleep, there were not connections inside the heart network (see Fig. 7). The REM sleep stage presented an important activity inside the brain network (see Fig. 8).

Observing each sleep stage, the adults group presented more connectionsw inside the brain network. This is seen when a contrast between figures 3 and 7 are analyzed. For the deep sleep stage, it is possible to see how the children group gets a greater interaction from the heart node, which can be noticed with the red arrows in figure 8. Finally, the children group also register a higher number of interactions in the brain network for the REM sleep stage, which can be compared to the adults group where there are just three connections to the δ subband.

Finally, the light and deep stages did not have significant differences for adults and children through the chi-square test.

## DISCUSSION

Our results indicate that the occurrence of these EEG and ECG networks might be attributed to the cardiovascular control in children, which is a higher order mechanism. Schwab et al. reported the coordination of the EEG and the HR of preterm neonates during NREM sleep^30^, finding that EEG bursts are associated with heart rate accelerations of the HR.

Analysis on children and adults data showed a reduction of 3% in REM stage sleep from children to adults groups. This effect has been consistently reported for more than 40 years^12,31^, showing a a reduction in the duration of this sleep stage, which mostly occurs during first 3 years, a period characterized by rapid growth of the connectivity and synapses^12^. We found a higher number of connections in REM stage sleep in the children group than in the adults group, and this difference may be related to the brain maturity process (Figs. 5 and 8).

Furthermore, it has been demonstrated that the NREM stage sleep δ subband has a decreasing activity during the adolescence, which is assumed to the synaptic pruning during this period of the life^12,32^. Liu et al. have reported that in deep and light sleep stages, the interchannel interactions are mainly mediated through the δ and α subbands, and that during deep sleep, the δ subband network is dominant whereas during light sleep, the δ subband network exhibits dominance^33^. Other studies addressed the slow waves relevance that complement this concept were reported by Jurysta in^34,35^.

Both groups in the present study showed high values considering the α subband as a target when the entire night was analyzed (Figs. 1 and 2). For the children group, this connection had a high frequency for the light sleep and REM sleep stages (note that the column that represents the target γ has the darkest squares, Figs. 6a and 8a). In the adults group, this γ effect was present for the light sleep stage only (Fig. 3a). These findings are consistent with published studies, in which γ subband connectivity has been demonstrated as a center of communication^17^. Liu et al. reported that during REM sleep in adults, the networks of all frequency subbands have comparable contributions to the analysis for communication between different brain areas, and exhibited slight prevalence of network interactions for the γ and α subbands, whereas the γ subband dominated during wakefulness^33^.

Our present findings of the brain wave network interactions and similar results from other studies indicate an evolution in the transition from one physiological state to another. This feature follows a particular pattern of reorganization, indicating a new aspect of neural plasticity, reflected in the coordinated activation of different brain rhythms across brain areas^16,17,33,36^.

For the children group, the entire night analysis (see Fig. 2) exhibited three sources with null connectivity. From VLF subsystem, there were not communications to HF, LF and α. The δ subband did not send information to LF and HF, and γ subband was not a source for the subsystems LF and β. In contrast with the adults group, the HF subsystem that did not emit information to the LF and β subsystems in the children group. There are no studies in preschool and school children that establish the normal pattern of these networks in this population. More studies are required in children of different ages to observe the evolution of these heart-brain networks.

A similar approximation from the analysis of parasympathetic nervous system activity and sleep was reported by El-Sheikh et al.^37,38^, where the respiratory sinus arrhythmia is argued as the “brake” of the heart rate in case of stress. At the same time, these respiratory sinus arrhythmia factors are associated to social parameters as marital conflicts and adjustment problems that can influence sleep in children. In the present study, the physiological network analysis of the heart and brain nodes may help to improve understanding of the influence of these factors from and to the brain network.

The importance of these findings is related to clinical applications in sleep-related disorders and autonomic regulation. For example, in obstructive sleep apnea, better understanding of the heart-brain networks during sleep could help form a paradigm of how a sleep breathing disorder can lead to permanent dysregulation of autonomic cardiovascular control, resulting in sustained sympathetic hyperactivity^39^. Previous studies have reported that a combination of cardiovascular and respiratory abnormalities associated with sleep disorders can increase cardiovascular risk. These abnormalities affect the control and regulation of breathing, manifesting disorders such as multiple system atrophy and congenital central hypoventilation syndrome^40^. In relation to this control, an understanding of normal HRV maturation in infants allows for reference values to be established for future studies of autonomic impairment in children^41^. Understanding this complex influence of the autonomic nervous system on the physiological dynamics of sleep can help to provide strategies of therapeutic approaches in children and adults^22^. Finally, a clinician perspective based on HRV information might be an useful tool for detecting autonomic irregularities associated to neurological disorders through the assessment of the brain–heart connections^39^.

The number of subjects we examined is a limitation of the study, and studies with larger group sizes are needed. A balanced participation of women and men is another aspect to take into account, especially in the adults group, where the sample considered only one male. Additionally, because the subjects were selected from a population referred to a sleep study, there are a limited number of “healthy” subjects, and it therefore cannot be ruled out that the subjects had another clinical condition, despite having a normal AHI. Finally, new techniques can provide more information for the analysis of EEG and HRV (for example, computations based on transfer entropy or neural networks Granger causality), which will be included in future studies.
